# Predicting pharmaceutical prices. Advances based on purchase-level data and machine learning

**DOI:** 10.1186/s12889-024-19171-9

**Published:** 2024-07-15

**Authors:** Mihály Fazekas, Zdravko Veljanov, Alexandre Borges de Oliveira

**Affiliations:** 1 Department of Public Policy, Central European University, Quellenstraße 51, 1100 Vienna, Austria; 2 World Bank, 1818 H Street, WA DC, 20433 USA

**Keywords:** Pharmaceutical products, Procurement, Machine learning, Health policy

## Abstract

**Background:**

Increased costs in the health sector have put considerable strain on the public budgets allocated to pharmaceutical purchases. Faced with such pressures amplified by financial crises and pandemics, national purchasing authorities are presented with a puzzle: how to procure pharmaceuticals of the highest quality for the lowest price. The literature explored a range of impactful factors using data on producer and reference prices, but largely foregone the use of data on individual purchases by diverse public buyers.

**Methods:**

Leveraging the availability of open data in public procurement from official government portals, the article examines the relationship between unit prices and a host of predictors that account for policies that can be amended nationally or locally. The study uses traditional linear regression (OLS) and a machine learning model, random forest, to identify the best models for predicting pharmaceutical unit prices. To explore the association between a wide variety of predictors and unit prices, the study relies on more than 200,000 purchases in more than 800 standardized pharmaceutical product categories from 10 countries and territories.

**Results:**

The results show significant price variation of standardized products between and within countries. Although both models present substantial potential for predicting unit prices, the random forest model, which can incorporate non-linear relationships, leads to higher explained variance (R^2^ = 0.85) and lower prediction error (RMSE = 0.81).

**Conclusions:**

The results demonstrate the potential of i) tapping into large quantities of purchase-level data in the health care sector and ii) using machine learning models for explaining and predicting pharmaceutical prices. The explanatory models identify data-driven policy interventions for decision-makers seeking to improve value for money.

**Supplementary Information:**

The online version contains supplementary material available at 10.1186/s12889-024-19171-9.

## Background

Countries around the globe continuously face difficult choices concerning the procurement of pharmaceutical products given opposing pressures of increasing costs, rising demands and budget constraints. Such challenges are particularly pressing in low- and middle-income countries where public budgets available for healthcare are more limited compared to high-income countries [1]. The COVID-19 pandemic has further stressed the already strained systems across the world. For example, health expenditures in the Latin America and the Caribbean (LAC^1^) region (3,8% of GDP) are lower compared to OECD (Organisation for Economic Cooperation and Development) countries (6,6% of GDP) which is compounded by higher levels of corruption, such as approximately 11% bribery rates in public health centres [2, 3].

The rapid increase in costs of pharmaceutical purchases contributes to the failure to provide equitable and quality healthcare. Therefore, there is a deep-seated need to better understand the drivers of pharmaceutical prices, so that governments can purchase the highest possible quality for the lowest possible price and be equipped with better tools for curbing corruption [4].

There is a large body of evidence in the literature that studies the association between pharmaceutical prices and the number of bidders that compete in tenders, the structure of the market [5], or (de-)centralization of the procurement process [6, 7]. To explore the effects of such predictors on prices, studies rely on measures such as price elasticity [8], relative prices of purchased goods, or simply on expert estimations and market-level average prices across countries [9]. The literature typically looks at specific pharmaceutical products [10], or only at advanced countries [9]. More sector-specific predictors in the literature that have been explored as determinants of pharmaceutical prices include patent expiration and generic status of the product and competition [11], the country’s transparency during procurement stages [12], the availability of open data [13], or production costs [14]. Nevertheless, the public health literature has largely neglected a host of administrative factors which have been shown to impact prices in different settings by the public procurement literature. These include the type of public procurement procedure used, the institutional capacities and qualities of the purchasing authorities, the design choices made during the preparation of the tender documentation, such as the length of the advertisement period or the month when the tender is launched [15], and the quantity of procured products [14]. As we will show, these factors explain a large portion of variation in drug prices; hence, their relative absence from the literature limits our understanding of drug price determinants.

In order to bridge this gap between the public health and the broader public procurement literature, the article investigates transaction- and organisation-level factors that influence pharmaceutical purchase prices across a wide set of countries. It uses micro-level public procurement data on over 200,000 purchases from 8 countries (Brazil (federal), Costa Rica, Ecuador, Mexico, Panama, Paraguay, Peru, and Uruguay) and 2 territories of Brazil (Amazonas and Santa Catarina). Based on such a high-granularity, large-scale public procurement database, we analyse and predict pharmaceutical prices. Taking advantage of the availability of data on the quantity and price of purchased goods, we calculate unit prices of standardised pharmaceutical products. Subsequently, we build models using a plethora of predictors identified in the literature to explore their effects on unit prices of pharmaceutical products.

The 8 countries and 2 territories were selected for the study based on i) comparability of purchasing systems; ii) availability of sufficiently high quality data, and iii) as balanced set of countries across LAC as possible. The availability of high quality micro-level data across multiple comparable, yet different countries and territories allows nuanced perspectives on regional variations and commonalities. Our aims are aligned with, for instance Steiner et al. [16] who show that there are similarities across all countries in the Americas regarding the national essential medicine lists. Such commonalities make it all the more important to investigate the causes of price variation even within narrowly defined and hence homogeneous drugs. Establishing and explaining large within-country price variations underline the importance for the public health literature to go beyond the national level and analyse prices at the individual purchase level.

## Methods and data sources

### Institutional context

While a comprehensive description of the drug acquisition policies and institutions in each country and territory studied is beyond the scope of this article, we provide a brief general background on the institutional context in order to establish the sources of variation in unit prices and procurement behaviours. Although national agencies can set ceilings or ranges for prices of certain pharmaceuticals and medicines [17], most public procurement takes place at decentralised levels, thus allowing for individual decisions and negotiations to influence unit prices within the national price framework if one exists for the particular drug[6, 7].^2^ As regional and local healthcare bodies and hospitals can procure individually, their decentralised decisions lead to considerable price variation even for the very same product. This is also confirmed by prior research: Vargas et al. [18] in their study on pharmaceuticals in the LAC region, note the significant variation of procurement prices across different jurisdictions that can be influenced by a wide range of factors like different market structures and diverse policies. Moreover, they further stress that substantial savings can be generated when tenders are aggregated across hospitals and primary healthcare centres.

All countries in our dataset have some forms of centralised procurement for drugs, however, centralised procurement is still underdeveloped and under-utilised [19, 28].^3^ Brazil as a federal state also enables decentralised procurement managed by the federal states. Here, drug purchasing is also conducted by decentralised public and health-related institutions. For instance, the federal share of drugs and medicines procurement in Brazil in 2019 represented only 16 percent [6]. Underlining the decentralised nature of drugs purchases, we have a large number of unique purchasing bodies^4^ within the datasets for each of our countries and territories (Table 1). Such diversity of individual purchasing decisions offers ample variation in public procurement practices which our models tap into for explaining prices.

**Table 1:** Distinct number of buyers per country/state

Country	Different buyers
Brazil (federal)	144
Amazonas	59
Santa Catarina	40
Costa Rica	30
Ecuador	2706
Mexico	83
Panama	439
Paraguay	68
Peru	127
Uruguay	200

### Data and indicators

In order to design a model that will enable us to predict pharmaceutical prices in the LAC region, we have relied on a unique dataset collected through web scraping of the public procurement websites of the procurement authorities or exported directly from government contract repositories. The final dataset contains structured information such as the procedure type used for the tendering process, the number of received bids, names of buyers and bidders, dates of tender notice, tender deadline, and tender award decision date.

The greatest challenge for developing a harmonised dataset that contains the same pharmaceutical products across all countries/territories and periods was matching product classifications. We relied on a semi-automated matching method with extensive manual crosschecks in order to match national product codes and descriptions to the United Nations Standard Products and Services Code (UNSPSC). Our matching strategy starts with the full dataset for each country and territory, including healthcare and non-healthcare data. Then we select all pharmaceutical-related observations using the national product classifications (Table 2). After this, we proceed with matching national product categories and descriptions to the standard UNSPSC classification. At the end, we only retain those observations which have a valid UNSPSC product code that overlap across all (or almost all) datasets. By implication, we removed all those observations which had missing product codes and descriptions. This filtering and matching process resulted in a considerable reduction of the full dataset from 789,183 contracts to 262,264 matched contracts using the UNSPSC scheme. Furthermore, due to incomplete information about the tender price or quantity of purchased goods, it was not possible to calculate the unit prices for a few observations. Therefore, these observations, which contained NA unit price values or in some cases unusually high/low unit prices, were removed from the dataset. Our final analysis dataset remains very large, encompassing 237,021 purchases for the period 2012–2021,^5^ containing 970 unique pharmaceutical products. These product categories range from ordinary pharmaceutical products (unbranded or branded, such as ibuprofen (UNSPSC code -51,142,106) or amoxicillin (UNSPSC code—51,101,511)) to more specialised originator products that are most often protected through various patents (e.g. some vaccines—poliovirus vaccine (UNSPSC code—51,201,616), measles and rubella virus vaccine (UNSPSC code—51,201,646)). Unfortunately, our data does not contain information on whether it is a generic drug or patented drug, or whether it was produced domestically or imported [17, 18].

**Table 2:** Dataset overview

*Country*	*Years*	*Full pharma datasets*	*UNSPSC matched dataset*	*Datasets used for the analysis*
*Amazonas (Brazil)*	2014–2018	19,938	3704	2629
*Brazil (federal)*	2014–2016	58,243	15,864	15,801
*Costa Rica*	2016–2017	724	724	720
*Ecuador*	2013–2017	453,329	186,214	186,214
*Mexico*	2012–2021	156,906	8914	2835
*Panama*	2014–2018	22,959	12,152	12,152
*Paraguay*	2012–2016	18,381	18,381	9590
*Peru*	2015	2971	1310	1271
*Santa Catarina (Brazil)*	2014–2017	29,515	11,525	2433
*Uruguay*	2014–2018	26,217	3476	3376
*Total*		789,183	262,264	237,021

The main dependent variable in the models is log unit price. Although using unit prices at the contract award is imperfect measurement (for instance it cannot factor in product quality), nonetheless, it approximates better value for money compared to other similar measurements, such as relative prices or price elasticity. The price calculated in our dataset and used for the analysis is the price paid by buyers directly to suppliers, i.e., the already discounted price compared to reference prices. We calculate our dependent variable using Eq. 1.

*Equation 1: Log unit price formula*

1 $$\log\left(unit\;prices\;at\;contract\;award\;\right)\;=\;\log\;\left(\frac{total\;value\;of\;items\;contracted}{s\tan dardised\;quantity\;of\;items\;contracted}\right)$$

**Fig. 1:**
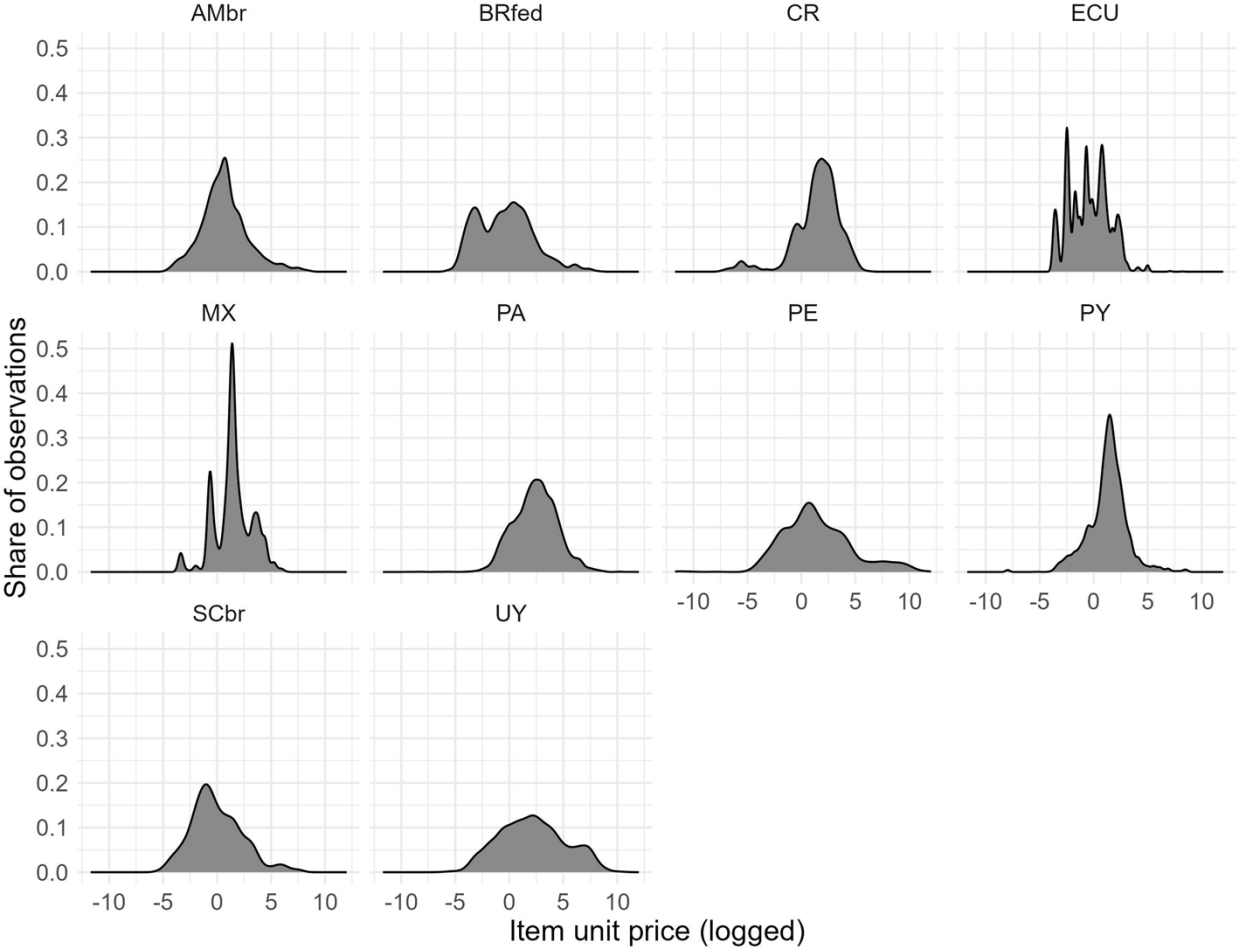
Log unit price distributions, by country, in USD

We take the natural logarithm of absolute unit prices so that price distributions for each product follow a distribution closer to normal. As expected in the literature, some purchasing authorities pay systematically more for standardised goods [19]. Figure 1 shows significant variations in prices for equivalent goods across and within countries. All prices are converted into USD using PPP (purchasing power parity) exchange rates and correcting for inflation.

To identify potential predictors of unit prices, our analysis relies on policy-relevant indicators in the healthcare, economic, and public administration literature. We have grouped the indicators into three broader categories: i) directly influenceable policies, such as procedure type [20, 21], advertisement period [22], month of spending [15], or product bundling [23]; ii) indirectly influenceable policies, such as number of bidders, supplier size, supplier specialisation, or supplier market share[24, 25], and iii) structural market conditions, such as buyer location (country/territory), year, and product code. While many of these indicators are continuous or integer in their original form, we transformed all of them into deciles plus a missing category. This allows for retaining records which have a missing value on one of the predictors but no missing on others. In addition, using deciles allows for considering non-linear relationships in the linear regression models too. Detailed explanations of the indicators, decile ranges and distributions are available in the Appendix. Table 3 presents an overview of all variables included in our models.

**Table 3:** Overview of indicators used in the analysis

Type	Variable name	Variable definition	Orig. variable type
Dependent variable	(log) unit price	Logarithm of unit price	Continuous
Market conditions	UNSPSC	Product codes of pharmaceutical products	Categorical
	Country/territory	Location of the public buyer	Categorical
	Year	Year of contract award	Categorical
Predictors: Directly influenceable	Quantity of purchased goods^a^	Number of units purchased	Continuous
	Product bundling	Number of different items purchased in the same procurement process	Integer
	Procedure type	Procedure type used in the tender (competitive, non-competitive, restricted, and NA)	Categorical
	Submission period	Number of days between the call for tender and bid deadline	Continuous
	Decision period per bids number	Average number of days per bid between bid deadline and award notice	Continuous
	Month	The month when the tender took place	Categorical
	Success rate	Rate of successfully concluded tenders over all tenders, by buyer and year	Continuous
Predictors: Indirectly influenceable	Number of bidders	The number of bidders participating in the tender	Integer
	Supplier market share	Annual share of the given supplier in the product market	Continuous
	Buyer’s spending concentration	The share of contract value that is awarded to the same supplier by the same buyer in a year	Continuous
	Supplier specialisation	Number of markets the company supplies	Integer
	Same location	Buyer and supplier from the same city	Categorical
	Supplier size	Size of the company based on total value of contracts won	Continuous

^a^Please note that the quantity of purchased goods is determined in the tender documentation by the buyer, which is followed by the stage of bid submissions of potential suppliers and bid evaluation by the buyer. The final price is determined by awarding the contract to the winning bidder, hence quantity decisions strictly precede price decisions, making the 2 variables distinct and limiting endogeneity bias

## Methods

Considering the uniquely wide scope of the compiled dataset – item-level pharmaceutical purchases -, we conduct both descriptive and explanatory analyses. The descriptive analysis aims to demonstrate the variation of unit prices across and within countries which underpin the added value of our high-granularity dataset over other approaches. Based on the theoretically identified predictors in the literature, we estimate and compare two models, a traditional regression method (Ordinary Least Squares) and a machine learning method (Random Forest) to investigate which offers a better explanation, that is a more precise price prediction [26].

The first modelling approach to predicting unit prices on the purchase level draws on all explanatory factors listed in Table 3 into a single Ordinary Least Squares regression model. This regression model^6^ includes fixed effects for country/state, year, and detailed product code (UNSPSC). The inclusion of such fixed effects accounts for unobserved heterogeneity not captured by observable factors and hence allows us to focus on the effects of predictors of interest. In particular, entering product code categories into the models implies that we explain price variations within each narrow product category, rather than across drugs. Considering the potential commonalities in price structures and their determinants across countries, we estimate coefficients for each predictor using the data on all countries and territories. Hence, they are best interpreted as average effects across the LAC region. Such a complex model allows for dataset-wide price predictions and simulation of hypothetical scenarios. Equation 2 specifies the regression model for log unit prices of standardised products on the level of individual items purchased:

*Equation 2: Linear regression model*

2 $$\Pr_{\mathrm i}\;=\;{\mathrm\alpha}_{\mathrm i}\;+\;\beta1\ast{\mathrm X}_{1\mathrm i}+\;\beta2\ast{\mathrm X}_{2\mathrm i}\;+\;\beta3\ast{\mathrm X}_{3\mathrm i}\;+\;\dots\;+\;\beta n\ast{\mathrm X}_{\mathrm{ni}}\;+\;{\mathrm\varepsilon}_{\mathrm I}$$

The second model we estimate is Random Forest (RF) using the same set of predictors as the regression model and running the estimation on the same level of observation (individual purchase).^7^ RF is an estimation based on intuitive tree-based models that sequentially split the sample into sub-samples to minimise prediction error. The model eventually aggregates over a large number of decision trees, whereby each tree is run using randomly varying parameters (random number of observations and predictors). We follow best practice by estimating RF models using 500 trees and 4 variables considered at each split (square root of the total number of variables) [27]. The advantage of the RF model lies in its ability to handle high-dimensional data and to estimate complex, non-linear, and interactive relationships without a priori defining the nature of such relationships. Parametric methods, such as standard linear models (i.e. OLS) require getting the functional form right for unbiased estimates. However, in our case, the potential number and types of interactions and non-linearities go far beyond what is feasible to accurately define based on theory.

To ensure comparability between the two models we use the same predictors for both models. Furthermore, for our models, we split our data into a train set and a test set using the 70–30 split rule. We train our models on the training dataset that contains 161,531 observations and predict the unit prices using the test set of 69,236 observations. Furthermore, we ensure that each country is split according to the same rule by employing a stratified train-test split.

## Results

First, we run a simple OLS model explaining the log unit price using a host of predictors (directly and indirectly influenceable by policy interventions) established in the literature. This arguably simple regression model explains 77 percent of total unit price variation (Table 4).^8^ The results of the regression model show that most of the predictors from the literature are significantly associated with unit prices as expected. Supplier market share is positively and significantly associated with unit prices, even though most of the positive effect comes from deciles 5 and 6, beyond which price impacts plateau or even slightly decrease. Overall, this indicates that the higher the share of certain suppliers in a market the higher the unit price of products. A similar direction and effect are expected and confirmed for buyer spending concentration, i.e., the more a buyer has its spending concentrated the higher the expected unit prices. Unlike the market-level predictor, whose effect on unit prices plateaus after the median, the estimated price impact of buyer’s concentration keeps on increasing.

**Table 4.**
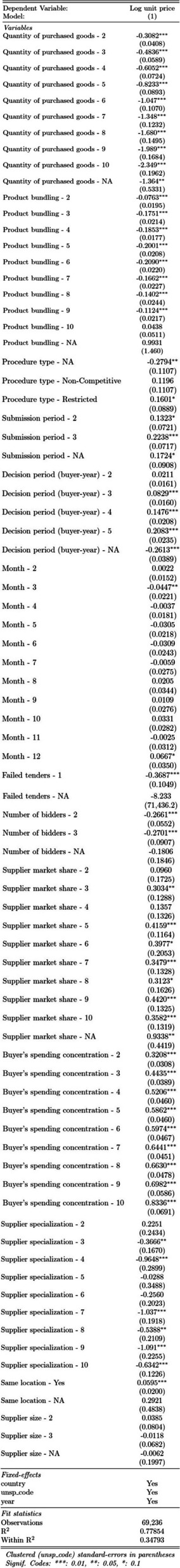
OLS regression results^a^

^a^Results from the training sample are available in the Appendix. The results show the same explanatory power of the model R2 = 0.77

**Fig. 2:**
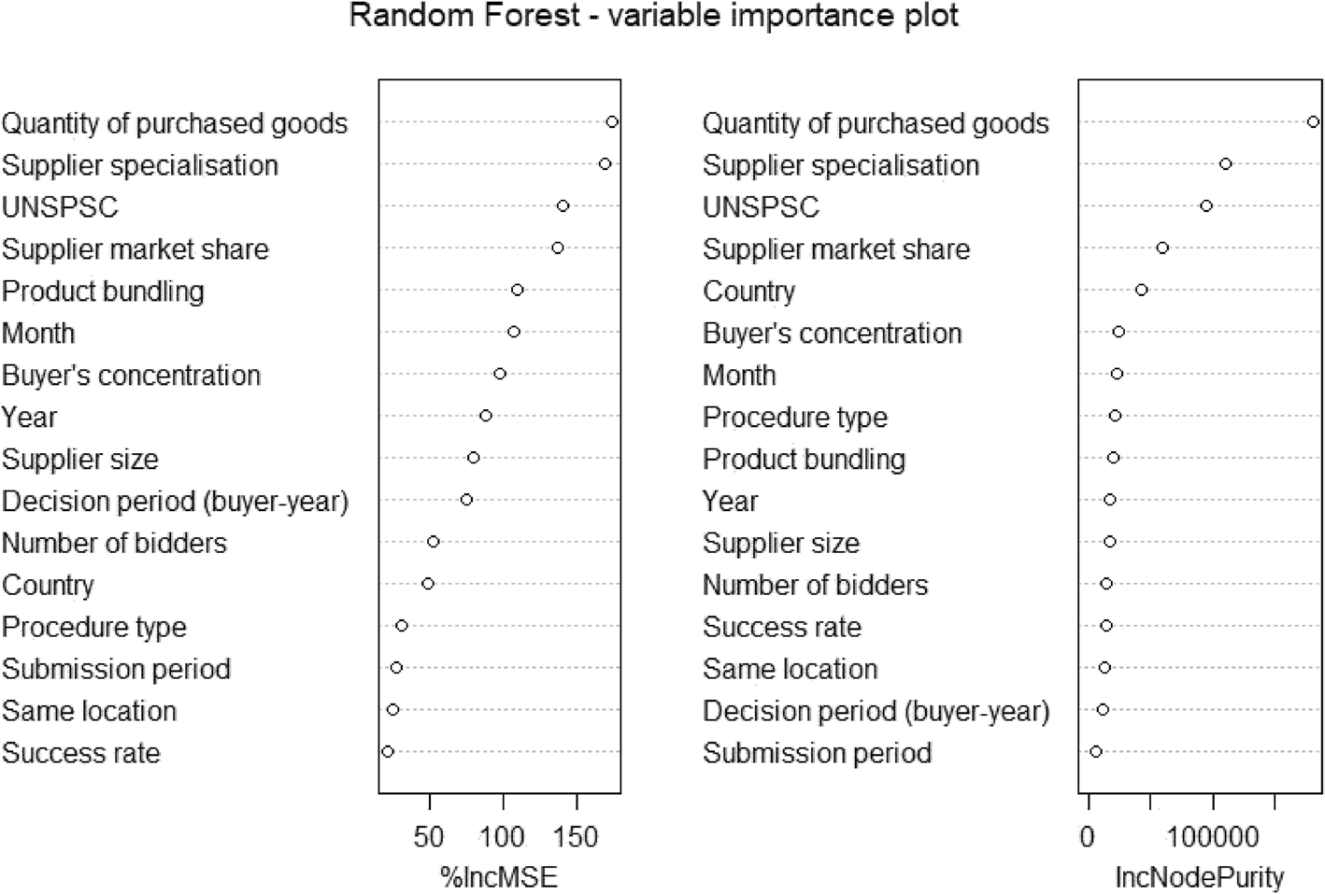
Random Forest—variable importance plot. Note: Quantity of purchased goods, Supplier specialisation, Supplier market share, Product bundling, and Buyer’s concentration are divided into 10 groups (deciles). Supplier size is divided into 3 groups (small, medium, and large companies). The submission period is divided into 3 groups (and an NA), and the decision period is divided into 5 groups/deciles

Predictors related to more indirectly influenceable policies, such as the number of bidders participating in tenders, are significantly and substantially associated with unit prices. The regression model indicates that the higher the number of bidders the lower the unit prices, while controlling for country, year, and product codes. Restricted procedures are associated with higher unit prices than open procedures. However, non-competitive procedures are insignificantly associated with higher unit prices. The size of the purchase (purchased quantity) and bundling different products together are both significant and substantial predictors of lower unit prices. Namely, each decile of both predictors tends to be negatively and significantly associated with unit prices.

From an organisational point of view, predictors such as the decision period (per buyer-year-item) and submission period also indicate that more efficient and better-organised organisations tend to pay less for their pharmaceuticals. Too long submission periods or too long decision periods could be related to inefficient or poorly organised procurement processes or simply a proxy for corrupt practices due to manipulating tenders. In a similar vein, the literature shows that badly executed procurement plans throughout the entire year could force buyers to spend their surplus budgetary funds in the last months of the financial year, and simultaneously drive the product prices up. Such end-of-the-year spending fever is to a certain extent confirmed by our regression model. December has a significant and positive association with higher unit prices (compared to January, which is the reference category).

Our second model is Random Forest (RF). In order to optimize price predictions, two meta-parameters had to be tuned, the number of trees (500 trees) and the number of variables to sample at each run (4 variables). Overall, the RF model outperforms the linear regression model in terms of explanatory power and prediction error. It accounts for 85 percent of the unit price variance. The advantage of the RF algorithm is that it can flexibly account for non-linear relationships without the researchers a priori specifying the nature of non-linearities. As we have seen in our linear regression model, some predictors such as supplier market share, product bundling, or supplier specialisation are estimated to have an inverted-U shape effect. It is precisely these predictors (along with the quantity of purchased goods) that are most important for RF model prediction accuracy and hence appear on the top of the variable importance plot (Fig. 2).^9^ The two metrics we use to understand variable importances and hence begin to interpret the RF models are i) the Increase in Mean Standard Error (IncMSE) and ii) decrease in node impurities (IncNodePurity) [27]. The large values of the two variables indicate that the predictors are important predictors for accurately estimating log unit prices. For instance, concerning quantity of purchased goods the IncMSE indicates how much the mean standard error of the model will increase if the values of the predictor quantity of purchased goods are randomly shuffled, and keep the rest of the variables the same. Node purity relates to the variable’s contribution to the purity of the terminal nodes of the RF trees, measured as residual sum of squares. Higher numbers indicate greater homogeneity and improvements of model predictive power.

**Fig. 3:**
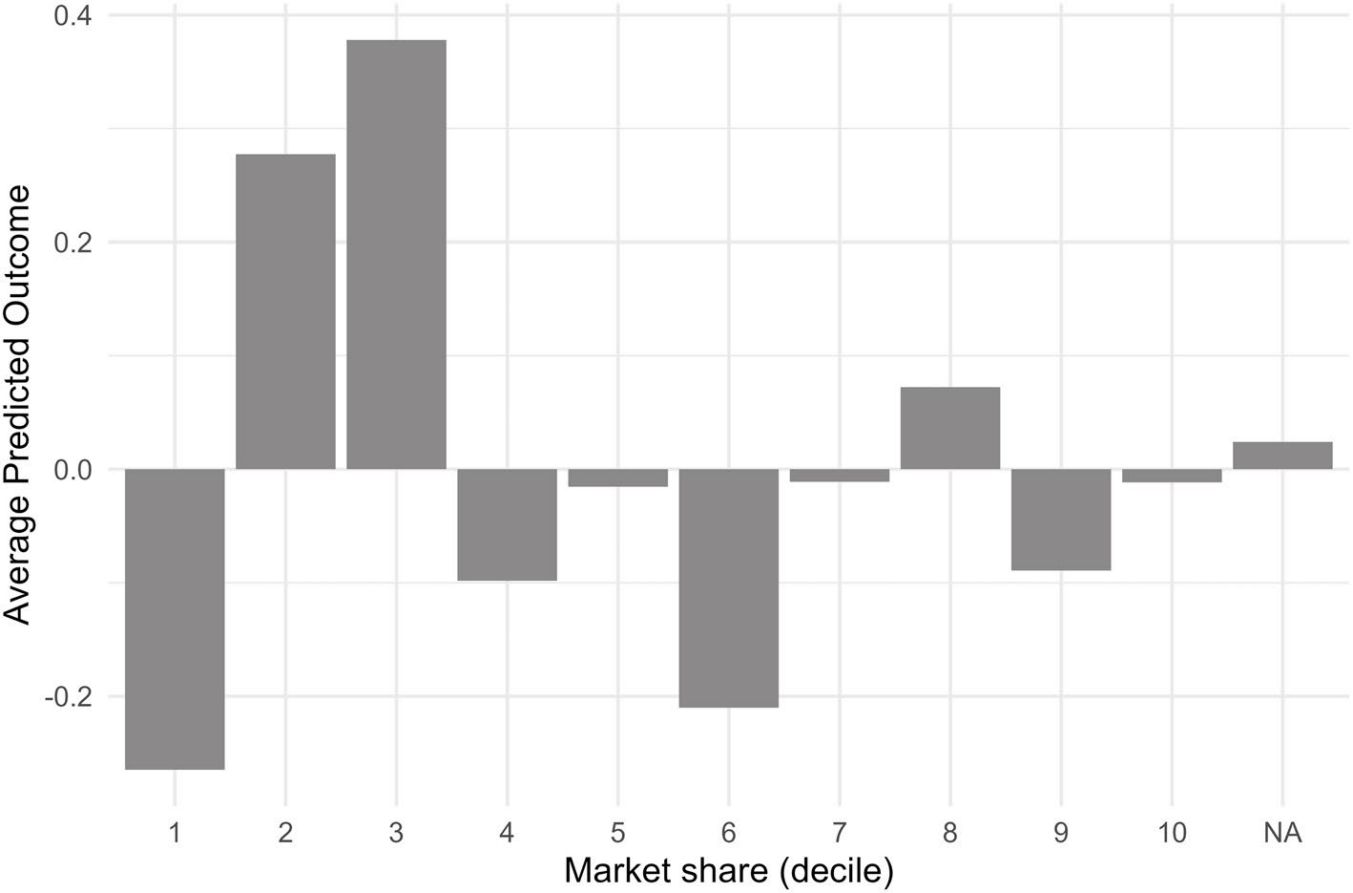
Partial dependence plot—supplier market share (deciles) and log unit price

To illustrate the importance of the top predictors and demonstrate their effect size and direction, we review one of them. Through supplier market share, we aim to capture the prevalence of monopolies and oligopolies at the supplier level. For instance, the partial dependence plot for supplier market share reveals a non-linear relationship with unit prices. The partial dependence plot visualises each decile’s predicted log unit price. Deciles 2 and 3—which imply lower annual market shares—have the highest predicted unit prices, while more concentrated markets—deciles 5, 7, 8 and 10—have somewhat lower but still comparatively high predicted unit prices (Fig. 3).

### Model comparisons

To compare the performance of the models, we first look at their prediction errors (Table 5). The linear regression model has a higher mean absolute error (0.746) and root mean squared error (1.001). The RF model performs substantially better concerning both measures. Its mean absolute error is more than one-third lower (0.459), while its root mean squared error is also substantially lower (0.809). Lastly, the explanatory power of the RF model is slightly higher compared to the linear model. Although both models explain a considerable part of the variation, the linear regression of 78 percent of explained variance is outperformed by the 84 percent explained variance of RF.

**Table 5:** Models comparison

	**Linear regression**	**Random Forest**
Mean Absolute Error (MAE)	0.75	0.46
Root Mean Squared Error (RMSE)	1.00	0.81
R^2^	0.77	0.85

## Discussion

The results of the present study, together with the findings in the literature, help to identify the likely price impact of certain factors to make better informed policy choices achieving lower pharmaceutical prices. Based on these models, analysts can formulate policy recommendations and interventions to achieve better value for money. Data-driven policy recommendations can identify specific procurement practices which are more costly, for example requiring or facilitating longer advertisement periods across the board. Moreover, administrative interventions can be targeted at inefficient public entities as flagged by the predictive models, for example holding public sector managers accountable for purchasing decisions. A major advantage of our predictive models is that they offer a clear prioritisation as to which procurement behaviours are worth influencing for better fiscal outcomes. Given the nature of the predictor variables, most recommendations and interventions will not require major legal or institutional changes, but rather individual entities’ adjustments through better practices and training.

Specifically, predictors in the analysis which can be directly influenced by policy offer a straightforward avenue for savings. Running public tenders through an open procedure stimulates competition, and as our results suggest lower unit prices. Although it is more time-consuming and complicated to implement open procedures for certain products, using this procedure represents a good predictor for lower unit prices. Furthermore, allowing for sufficient time concerning the tender advertisement period provides potential bidders with an opportunity to better prepare. Avoiding too many tenders in December, compared to January, is associated with lower unit prices. Although not all months are significant predictors, borrowing from the literature end-of-the-year spikes (spending in the last few months of the fiscal year) contribute to increased unit prices. Additionally, in the RF model, month performs quite well as an important predictor for unit prices. Therefore, spreading such tenders throughout the year can result in significantly lower prices. Similar improvements can also be obtained by improving the organisational efficiency of the public buyers by focusing on more expedient decision-making.

Considering indirectly policy influenceable predictors, which are harder to influence through policy shifts, we find that more intense competition contributes to lower unit prices. First, the number of bidders that submit offers can be considered as a proxy for greater competition. Both bidder categories, which denote more than 2 bidders, are associated with significantly lower unit prices. Stimulating greater competition, for example, by providing training for buyers to better prepare tenders, can substantially reduce prices. Second, diversifying supply markets pays off. Better value for money is expected when concentration is lower at buyer or market levels. Therefore, government policies should target tenders from the most concentrated deciles to diversify and move into less concentrated and lower deciles. Third, awarding tenders from the same location as the buyer contributes to further increasing unit prices. There is additional room for identifying and implementing policies that encourage the participation of bidders from other regions. Such policies can indirectly stimulate competition and eventually lead to lower prices.

### Limitations

Although the size of our dataset and the corresponding spatial and temporal scope provide a substantial sample to pursue our research goals, some limitations remain. First, Table 2 shows that our sample is not well balanced across countries, leaving space for improving the dataset by additional data collection and better processing. The most evident gap is the small-matched set of products from many datasets, especially from the Mexican data, which has substantially reduced our initial sample of pharmaceutical contracts. Some countries, such as Costa Rica and Peru, can also be expanded to include further years. Second, some potentially impactful variables are missing from the analysis: Factors that are more difficult to measure and quantify, such as the quality of products and the brand of the product, or some qualitative aspects such as negotiation strategies. Nevertheless, despite these limitations, the models explain substantial variation in pharmaceutical unit prices. Third, the dependent variable, unit price of standardised drugs, may not adequately reflect actual prices paid. If suppliers offer informal discounts or if delivery is deficient without the buyer recording it, our unit price measure will be biased. Further research could look into payments and deliveries data to complement our contracting data.

## Conclusions

This article built explanatory models with high accuracy in predicting pharmaceutical prices in Latin America and the Caribbean region. The results show a promising avenue for using machine learning algorithms to predict unit prices of pharmaceutical products. Compared to a standard linear model (i.e. OLS), the Random Forest model accounts for a higher portion of total price variation and it has lower error values, both mean absolute error and root mean squared error. Furthermore, both models have confirmed the importance of the already established predictors in the literature.

The article makes at least three sets of contributions. First, the evidence presented in this paper suggests that the three sets of predictors (factors directly influenceable by policy, factors indirectly influenceable by policy, and structural market conditions) show promising pathways for policymakers to explore for providing better value for money in the procurement of pharmaceutical products. Directly influenceable factors, such as modifying the type of procedure or providing sufficient time for bidders to prepare could be more easily and readily achieved. However, with better planning and improvements in competitiveness, authorities could also achieve substantial improvements in policies such as stimulating greater bidder participation or diversifying procurement from suppliers from other regions. Both predictors are associated with lower unit prices. Lastly, at a structural level, better scheduling, i.e., preventing rush procurements in the last months of the fiscal year (avoiding the end-of-year spikes) can contribute to better expenditure. The second contribution of the paper shows the opportunity that researchers can take with using machine learning algorithms in predicting pharmaceutical prices. Third, our analysis has both identified and explained a large price variation within countries regarding the very same, standardised products. This level of price variation has been under-studied in the literature which should be alleviated in the future.

### Supplementary Information

Supplementary Material 1: Additional materials. Appendix with further technical details of the data and indicators used.

## Data Availability

Data collected from publicly available data sources for Brazil (federal) Ecuador, Mexico, Paraguay, Peru, and Uruguay can be accessed at the open repository maintained by the Government Transparency Institute: https://www.govtransparency.eu/gtis-global-government-contracts-database/. The other datasets (Brazil subnational datasets, Costa Rica, and Panama) were received directly from the governments under conditions of non-disclosure, hence those datasets cannot be deposited.

## Abbreviations

GDP: Gross Domestic Product
LAC: Latin America and the Caribbean
MAE: Mean Absolute Error
OECD: Organization for Economic Cooperation and Development
OLS: Ordinary least squared
PPP: Purchasing power parity
RF: Random Forest
RMSE: Root Mean Square Error
UNSPSC: United Nations Standard Product and Services Code

## Footnotes

1 Full list of abbreviations is available at the end of the paper.

2 However, given weak enforcement capacity in many of our countries and territories, we cannot rule out that some price ceilings and ranges are ignored by individual purchasers leading to even larger price variation than wound have been the case.

3 In our full dataset the share of central procurement agencies ranges from 0.01% in Panama to 0.62% in Uruguay and 0.98% in the state of Santa Catarina.

4 Some countries have fewer buyers, but the numbers are dependent on the size of sample and data standardisation.

5 Time period covered differs by country, with Mexico having the most years, ranging from 2012 to 2021.

6 We use the fixest package in R (https://cran.r-project.org/web/packages/fixest/index.html).

7 We use the randomForest package in R (https://cran.r-project.org/web/packages/randomForest/index.html).

8 Regression diagnostics and assumption checks can be found in the Annex, Figure A1 and A2; and Table A14.

9 A further comparative test, Delta R2, is available in the Appendix. It estimates the additional explanatory power of each predictor of the OLS regression on top of the baseline model. Specifically, Delta R2 shows how much more additional variance can be explained with each additional predictor on the top of the base model, which will be the con
