# Correction: Predicting pharmaceutical prices. Advances based on purchase-level data and machine learning

**DOI:** 10.1186/s12889-024-19701-5

**Published:** 2024-08-15

**Authors:** Mihály Fazekas, Zdravko Veljanov, Alexandre Borges de Oliveira

**Affiliations:** 1 Department of Public Policy, Central European University, Quellenstraße 51, 1100 Vienna, Austria; 2 World Bank, 1818 H Street, WA DC, 20433 USA

**BMC Public Health (2024) 24:1888**

10.1186/s12889-024-19171-9

The original publication of this article contained an incorrect funding section. The incorrect and correct information is listed in this correction article, the original article has been updated.

**Funding**

No funding received for this article.

**Funding**

The publication of this research was made possible by the CEU Open Access Fund.

