# Supplementary material for: Predicting pharmaceutical prices. Advances based on purchase-level data and machine learning
Source: BMC Public Health. 2024 Jul 15;24:1888. doi: 10.1186/s12889-024-19171-9 (PMC11247880; doi:10.1186/s12889-024-19171-9)
Supplement: Supplementary file 1 — Supplementary Material 1: Additional materials. Appendix with further technical details of the data and indicators used. [file 12889_2024_19171_MOESM1_ESM.docx]

# Appendix

## Definition of predictors

- **Quantity of purchased goods** - Buying in bulk, theoretically, should lead to product discounts and subsequently lower prices. Consolidating the demand by reducing the number of procurements with small purchases of one or few units should lead to better value for money.

Table A1: Quantity of purchased goods (deciles and ranges)

| **Quantity (purchased items deciles)** | **Minimum (items)** | **Mean (items)** | **Maximum (items** |
| --- | --- | --- | --- |
| Decile 1 | 1 | 11 | 48 |
| Decile 2 | 48 | 91 | 150 |
| Decile 3 | 150 | 233 | 300 |
| Decile 4 | 300 | 468 | 600 |
| Decile 5 | 600 | 891 | 1104 |
| Decile 6 | 1104 | 1672 | 2160 |
| Decile 7 | 2160 | 3234 | 4600 |
| Decile 8 | 4600 | 6549 | 10000 |
| Decile 9 | 10000 | 15222 | 25000 |
| Decile 10 | 25000 | 165825 | 75644900 |

- **Product bundling** - Procuring too many (different) items within the same procurement process could result in much higher prices, mostly in order to save time by avoiding paperwork for different procurements. Better strategic packaging of products of similar nature theoretically should result in more efficient processes and prices. Excessive number or too few items, conversely, results in higher prices.

Table A2: Product bundling (Average number of items per decile)

| **Product bundling (decile)** | **Average number of packaged items per decile** |
| --- | --- |
| Decile 1 | 235.3 |
| Decile 2 | 668.6 |
| Decile 3 | 1596.1 |
| Decile 4 | 2680.2 |
| Decile 5 | 3807.1 |
| Decile 6 | 4974.8 |
| Decile 7 | 6143.9 |
| Decile 8 | 7486.5 |
| Decile 9 | 9532.6 |
| Decile 10 | 1338925 |
| Decile NA | 3362.5 |

- **Procedure type** relates to the process through which purchasing authorities, such as national or regional authorities or public bodies, purchase goods, services or works. We distinguish between open procedure, as the most transparent procurement method, non-competitive procedures that can take the form of outright or direct award, negotiated without publication, and restricted procedure types.

Table A3: Procedure type

| **Procedure type** | **Number of observations** |
| --- | --- |
| Competitive | 201747 |
| Non-competitive | 12618 |
| Restricted | 12044 |
| NA | 10612 |

- **Submission period** – suppliers offer lower prices when given reasonable time to prepare and submit bids. Very short periods of tender advertisement for bids submissions could prevent better competition.

Table A4: Submission period (categories and ranges)

| **Submission period (deciles)** | **Minimum (days)** | **Mean (days)** | **Maximum (days)** |
| --- | --- | --- | --- |
| Submission period 1 | 1 | 4.1 | 7 |
| Submission period 2 | 7 | 10.7 | 15 |
| Submission period 3 | 15 | 37.8 | 286 |

- **The average organisational decision-making speed** predictor aims to assess the government agencies' organizational capacities, in terms of procurement and technical capacities. Agencies that take longer to evaluate bids tend to pay higher prices. More efficiently organized agencies process bids evaluation and award decisions faster.

Table A5: Decision-making speed (Average number per year-buyer)

| **Decision-making speed (per year-per buyer) - deciles** | **Average per agency** |
| --- | --- |
| Decile 1 | 190.5 |
| Decile 2 | 291.2 |
| Decile 3 | 468.4 |
| Decile 4 | 672.4 |
| Decile 5 | 895.9 |
| Decile NA | 367.4 |

- **Month** - ​Month relates to the importance of seasonality in procurement. It aims to capture the end-of-year spikes in procurements that can be more expensive, rushed and use less competitive procedures. It is defined from the publication first call for tender, i.e., when the call for tender was announced.

Table A6: Month

| Month | Number of observations |
| --- | --- |
| 1 | 19295 |
| 2 | 35018 |
| 3 | 17433 |
| 4 | 19538 |
| 5 | 21493 |
| 6 | 18726 |
| 7 | 23136 |
| 8 | 13534 |
| 9 | 21307 |
| 10 | 18473 |
| 11 | 20762 |
| 12 | 8306 |

- **Success rate** - Rate of successful tenders by buyer-year, transformed into a categorical variable with a cut-point of 0.7 (scale 0-1). 1 denotes tenders as successful.
- **Number of bidders** - Higher number of bidders that participate in the tender processes stimulate competition and consequently more competitive (and potentially) lower unit prices. Although procedure types are open, unless they are accompanied with a higher number of bidders, the unit prices could remain unchanged. When on average at least three bidders are participating, it leads to significantly lower unit prices.

Table A7: Number bidders (categories and ranges)

| **Number of bids (deciles)** | **Minimum (bidders)** | **Mean (bidders)** | **Maximum (bidders)** |
| --- | --- | --- | --- |
| Decile 1 | 0 | 1.2 | 2 |
| Decile 2 | 2 | 3.3 | 5 |
| Decile 3 | 5 | 12.9 | 75 |

- **Market share**- Monopolies and oligopolies of public procurement have an effect on the level of prices. More concentrated markets with one or a few suppliers (higher deciles) tend to be more expensive. Developing strategies for breaking up such monopolistic or oligopolistic behavior, where contracts are awarded on very concentrated markets should lead to lower unit prices.

Table A8: Market share (ranges per decile)

| **Market share (decile)** | **Minimum (%)** | **Mean (%)** | **Maximum (%)** |
| --- | --- | --- | --- |
| Decile 1 | 0.0000014 | 4.31 | 81.14 |
| Decile 2 | 0.036 | 17.97 | 100 |
| Decile 3 | 0.13 | 34.54 | 100 |
| Decile 4 | 0.37 | 50.58 | 100 |
| Decile 5 | 0.94 | 59.75 | 100 |
| Decile 6 | 2.75 | 73.05 | 100 |
| Decile 7 | 6.58 | 86.52 | 100 |
| Decile 8 | 10.99 | 89.91 | 100 |
| Decile 9 | 19.29 | 93.22 | 100 |
| Decile 10 | 36.37 | 97.25 | 100 |

- **Buyer’s concentration** – government agencies that showed a profile of monopolistic or oligopolistic tendencies in markets pay higher prices. Such profiles exist at the level of agencies even for markets that are competitive in public procurement. Breaking up such monopolies (the higher deciles) should lead to lower unit prices.

Table A9: Buyer’s concentration (ranges per decile)

| **Buyer’s concentration (deciles)** | **Minimum (%)** | **Mean (%)** | **Maximum (%)** |
| --- | --- | --- | --- |
| Decile 1 | 0.0000012 | 0.07 | 0.28 |
| Decile 2 | 0.0018 | 0.30 | 1.47 |
| Decile 3 | 0.0058 | 0.73 | 5.28 |
| Decile 4 | 0.012 | 1.52 | 16.44 |
| Decile 5 | 0.019 | 2.71 | 30.96 |
| Decile 6 | 0.033 | 3.95 | 37.37 |
| Decile 7 | 0.053 | 5.76 | 69.85 |
| Decile 8 | 0.084 | 8.25 | 87.73 |
| Decile 9 | 0.10 | 11.47 | 94.34 |
| Decile 10 | 0.53 | 21.56 | 100 |

- **Supplier specialization** - Highly specialized suppliers, i.e., those suppliers that offer only a few types of products, do this at a higher price. Some of these suppliers orient towards selling to the government through non-competitive procedures. Lower deciles represent more specialized suppliers.

Table A10: Supplier specialization (ranges per decile)

| **Supplier specialization (decile) items** | **Minimum (number of markets)** | **Mean (number of markets)** | **Maximum (number of markets)** |
| --- | --- | --- | --- |
| Decile 1 | 1 | 25.2 | 46 |
| Decile 2 | 46 | 56.5 | 62 |
| Decile 3 | 62 | 67.1 | 70 |
| Decile 4 | 70 | 81.2 | 88 |
| Decile 5 | 88 | 90.1 | 94 |
| Decile 6 | 94 | 101 | 109 |
| Decile 7 | 109 | 112 | 116 |
| Decile 8 | 116 | 117 | 119 |
| Decile 9 | 119 | 125 | 132 |
| Decile 10 | 132 | 143 | 349 |

- **Same location -** are the suppliers located in the same locational unit (city) as the buyer. Transformed into a binary variable, where 1 denotes buyers and suppliers are from the same location. Missing locations of the suppliers were classified as NA.

Table A11: Same location

| **Same location** | **Number of observations** |
| --- | --- |
| 0 (non-local suppliers) | 153501 |
| 1 (local suppliers) | 67739 |
| NA | 15781 |

- **Supplier size -** size of suppliers based on the total value of contracts received for the analyzed period. Based on their value, suppliers are split into 3 categories (small - 1, medium - 2, and large - 3).

Table A12: Supplier size

| **Same location** | **Number of observations** |
| --- | --- |
| 1 (small) | 18984 |
| 2 (medium) | 87965 |
| 3 (large) | 116621 |

## Regression diagnostics and assumption checks

Table A13: OLS regression results, on the training set

| Dependent Variable: | Log unit price |
| --- | --- |
| Model: | (1) |
| *Variables* |  |
| Quantity of purchased goods - 2 | -0.3420*** |
|  | (0.0441) |
| Quantity of purchased goods - 3 | -0.5282*** |
|  | (0.0619) |
| Quantity of purchased goods - 4 | -0.6486*** |
|  | (0.0727) |
| Quantity of purchased goods - 5 | -0.8823*** |
|  | (0.0898) |
| Quantity of purchased goods - 6 | -1.074*** |
|  | (0.1078) |
| Quantity of purchased goods - 7 | -1.395*** |
|  | (0.1252) |
| Quantity of purchased goods - 8 | -1.723*** |
|  | (0.1475) |
| Quantity of purchased goods - 9 | -2.070*** |
|  | (0.1664) |
| Quantity of purchased goods - 10 | -2.388*** |
|  | (0.1974) |
| Quantity of purchased goods - NA | -0.6078 |
|  | (0.4324) |
| Product bundling - 2 | -0.0645*** |
|  | (0.0148) |
| Product bundling - 3 | -0.1968*** |
|  | (0.0171) |
| Product bundling - 4 | -0.1886*** |
|  | (0.0171) |
| Product bundling - 5 | -0.1893*** |
|  | (0.0174) |
| Product bundling - 6 | -0.2043*** |
|  | (0.0203) |
| Product bundling - 7 | -0.1564*** |
|  | (0.0201) |
| Product bundling - 8 | -0.1499*** |
|  | (0.0240) |
| Product bundling - 9 | -0.1069*** |
|  | (0.0234) |
| Product bundling - 10 | 0.0711 |
|  | (0.0512) |
| Product bundling - NA | 0.7545 |
|  | (0.5244) |
| Procedure type - NA | -0.2319** |
|  | (0.0934) |
| Procedure type - non-competitive | 0.1435 |
|  | (0.0947) |
| Procedure type - restricted | 0.2218** |
|  | (0.0894) |
| Submission period - 2 | 0.1268** |
|  | (0.0520) |
| Submission period - 3 | 0.2249*** |
|  | (0.0570) |
| Submission period - NA | 0.1469* |
|  | (0.0785) |
| Decision period (buyer-year) - 2 | 0.0175* |
|  | (0.0106) |
| Decision period (buyer-year) - 3 | 0.0791*** |
|  | (0.0130) |
| Decision period (buyer-year) - 4 | 0.1372*** |
|  | (0.0153) |
| Decision period (buyer-year) - 5 | 0.2001*** |
|  | (0.0208) |
| Decision period (buyer-year) - NA | -0.2603*** |
|  | (0.0374) |
| Month - 2 | -0.0122 |
|  | (0.0149) |
| Month - 3 | -0.0263 |
|  | (0.0175) |
| Month - 4 | 0.0135 |
|  | (0.0171) |
| Month - 5 | -0.0245 |
|  | (0.0157) |
| Month - 6 | -0.0207 |
|  | (0.0214) |
| Month - 7 | -0.0157 |
|  | (0.0229) |
| Month - 8 | -0.0064 |
|  | (0.0260) |
| Month - 9 | 0.0208 |
|  | (0.0235) |
| Month - 10 | 0.0124 |
|  | (0.0234) |
| Month - 11 | 0.0036 |
|  | (0.0262) |
| Month - 12 | 0.0931*** |
|  | (0.0298) |
| Success rate - 1 | -0.6146*** |
|  | (0.0949) |
| Success rate - NA | -6.403 |
|  | (54,292.6) |
| Number of bidders - 2 | -0.2733*** |
|  | (0.0446) |
| Number of bidders - 3 | -0.2213*** |
|  | (0.0840) |
| Number of bidders - NA | -0.1373 |
|  | (0.1696) |
| Supplier market share - 2 | 0.0911 |
|  | (0.1787) |
| Supplier market share - 3 | 0.3052** |
|  | (0.1353) |
| Supplier market share - 4 | 0.1705 |
|  | (0.1230) |
| Supplier market share - 5 | 0.4328*** |
|  | (0.1117) |
| Supplier market share - 6 | 0.4335** |
|  | (0.1969) |
| Supplier market share - 7 | 0.3775*** |
|  | (0.1247) |
| Supplier market share - 8 | 0.3285** |
|  | (0.1606) |
| Supplier market share - 9 | 0.4900*** |
|  | (0.1234) |
| Supplier market share - 10 | 0.3499*** |
|  | (0.1258) |
| Supplier market share - NA | 0.0445 |
|  | (0.3821) |
| Buyer's spending concentration - 2 | 0.2907*** |
|  | (0.0276) |
| Buyer's spending concentration - 3 | 0.4106*** |
|  | (0.0372) |
| Buyer's spending concentration - 4 | 0.4918*** |
|  | (0.0407) |
| Buyer's spending concentration - 5 | 0.5641*** |
|  | (0.0460) |
| Buyer's spending concentration - 6 | 0.5834*** |
|  | (0.0428) |
| Buyer's spending concentration - 7 | 0.5942*** |
|  | (0.0429) |
| Buyer's spending concentration - 8 | 0.6352*** |
|  | (0.0477) |
| Buyer's spending concentration - 9 | 0.6578*** |
|  | (0.0572) |
| Buyer's spending concentration - 10 | 0.7915*** |
|  | (0.0682) |
| Supplier specialization - 2 | 0.1772 |
|  | (0.2512) |
| Supplier specialization - 3 | -0.3825** |
|  | (0.1661) |
| Supplier specialization - 4 | -0.9833*** |
|  | (0.2830) |
| Supplier specialization - 5 | -0.0352 |
|  | (0.3366) |
| Supplier specialization - 6 | -0.2774 |
|  | (0.1973) |
| Supplier specialization - 7 | -1.052*** |
|  | (0.1852) |
| Supplier specialization - 8 | -0.5900*** |
|  | (0.2126) |
| Supplier specialization - 9 | -1.093*** |
|  | (0.2210) |
| Supplier specialization - 10 | -0.6915*** |
|  | (0.1150) |
| Same location - 1 | 0.0667*** |
|  | (0.0182) |
| Same location - NA | -0.2808 |
|  | (0.3918) |
| Supplier size - 2 | 0.0135 |
|  | (0.0773) |
| Supplier size - 3 | -0.0248 |
|  | (0.0634) |
| Supplier size - NA | -0.1470 |
|  | (0.1124) |
| *Fixed-effects* |  |
| country | Yes |
| UNSPSC | Yes |
| year | Yes |
| *Fit statistics* |  |
| Observations | 161,531 |
| R2 | 0.77281 |
| Within R2 | 0.34542 |
| *Clustered (by UNSPSC) standard-errors in parentheses* | |
| *Signif. Codes: ***: 0.01, **: 0.05, *: 0.1* | |

Figure A1: Regression residuals


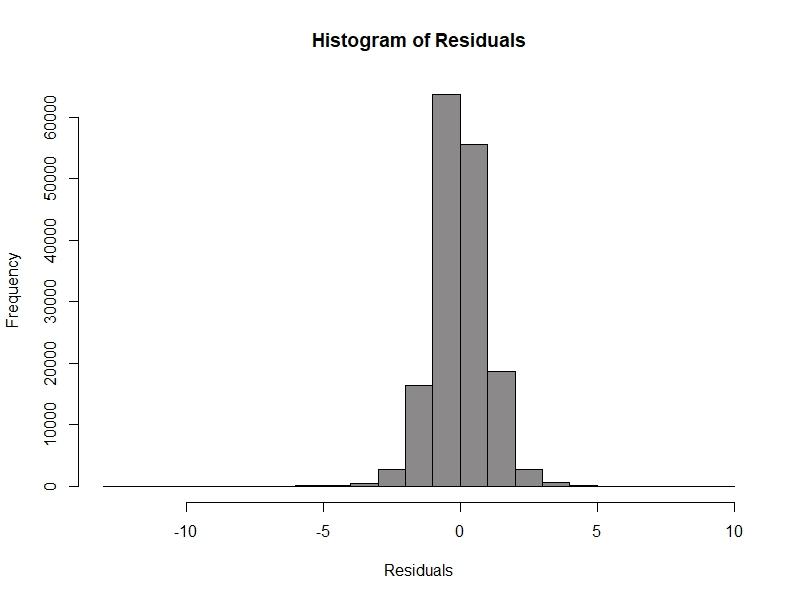


Figure A2: Homoscedasticity test


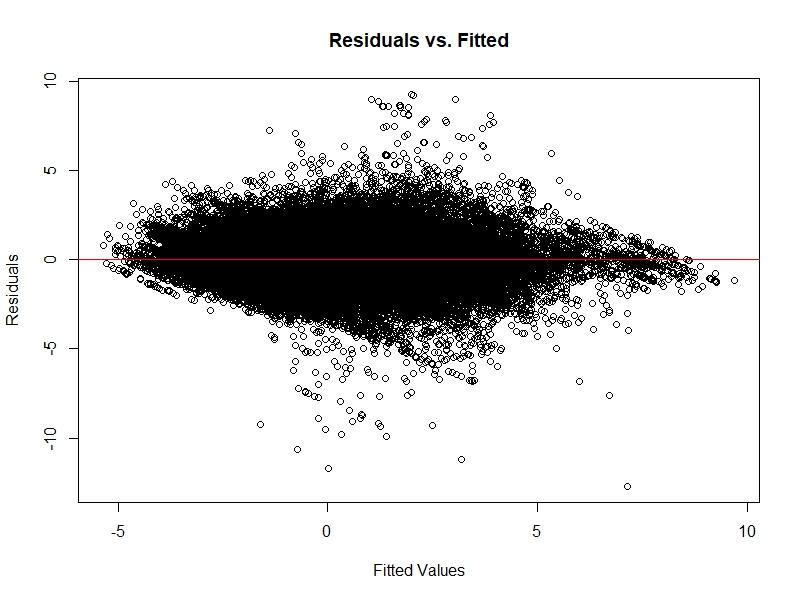


Table A14: Durbin-Watson test to check for autocorrelation

| **Test** | **Statistic** | **P-value** | **Conclusion** |
| --- | --- | --- | --- |
| Durbin-Watson | 1.9935 | 0.096 | No significant autocorrelation |

Figure A3: Marginal contribution based on Delta R2 and Random Forest variable importance plot


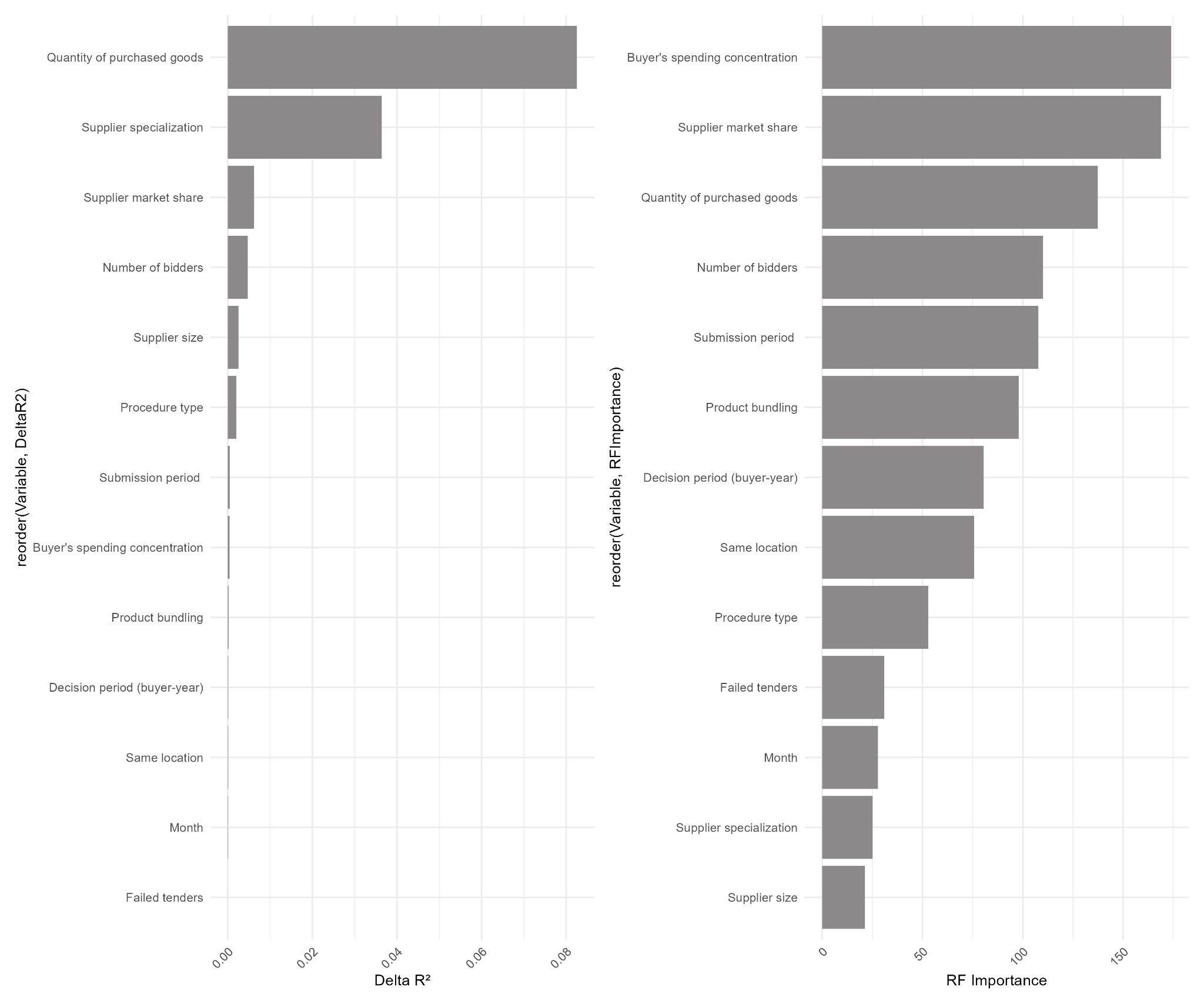


Table A14: Price variation across item quantity groups for Ibuprofen

| Item quantity (decile) | UNSP_code | Mean price (log) | Standard deviation (log) |
| --- | --- | --- | --- |
| 1 | Ibuprofen | 1.023 | 1.708 |
| 2 | Ibuprofen | 0.842 | 0.734 |
| 3 | Ibuprofen | 0.731 | 0.852 |
| 4 | Ibuprofen | 0.670 | 0.992 |
| 5 | Ibuprofen | 0.435 | 1.199 |
| 6 | Ibuprofen | 0.222 | 1.370 |
| 7 | Ibuprofen | -0.555 | 1.576 |
| 8 | Ibuprofen | -1.472 | 1.426 |
| 9 | Ibuprofen | -2.137 | 0.790 |
| 10 | Ibuprofen | -2.384 | 0.537 |

## Links to Public Procurement portals

Amazonas –<https://www.e-compras.am.gov.br/publico/>

Brazil –<https://antigo.comprasgovernamentais.gov.br/>

Costa Rica –<https://www.cgr.go.cr/01-cgr-transp/procesos-contrat.html>

Ecuador -<https://portal.compraspublicas.gob.ec/sercop/>

Mexico -<https://compranet.hacienda.gob.mx>

Panama -<https://www.panamacompra.gob.pa/Inicio/#/>

Paraguay -<https://www.contrataciones.gov.py/>

Peru -<https://www.gob.pe/7324-acceder-al-sistema-electronico-de-contrataciones-del-estado-seace>

Santa Catarina –<https://www.portaldecompras.sc.gov.br/#/>

Uruguay –<https://www.gub.uy/agencia-reguladora-compras-estatales/>
